# Editorial: Role of ion channels and metabotropic receptors in oligodendrogliogenesis: novel targets for demyelinating pathologies

**DOI:** 10.3389/fncel.2024.1517363

**Published:** 2024-11-12

**Authors:** Federica Cherchi, Matthew Swire, Davide Lecca

**Affiliations:** ^1^Department of Neuroscience, Psychology, Drugs and Child Health Area, Università degli Studi di Firenze, Florence, Italy; ^2^Wolfson Institute for Biomedical Research, University College London, London, United Kingdom; ^3^Department of Pharmaceutical Sciences, Università degli Studi di Milano, Milan, Italy

**Keywords:** oligodendrocytes, oligodendrocyte progenitor cells, G protein-coupled receptors, ion channels, miRNA, pharmacology, multiple sclerosis

Oligodendrocytes, the myelin-producing cells of the central nervous system (CNS), play a crucial role in facilitating rapid signal transmission and providing metabolic support to neurons. These cells arise from oligodendrocyte progenitor cells (OPCs), which are not only prevalent in the developing CNS, but persist into adulthood, comprising ~5–8% of total CNS cells (Levine et al., 2001). After CNS injury, such as in demyelinating diseases, OPCs are vital for generating new oligodendrocytes to remyelinate denuded axons. However, this regenerative process often fails with aging and chronic conditions, leading to irreversible axonal loss and subsequent neurological decline.

A growing body of evidence points to ion channels and metabotropic receptors as key regulators of OPC behavior influencing processes such as migration, proliferation, differentiation, and ultimately myelination (Bergles et al., 2000; Cherchi et al., 2021a; Krasnow and Attwell, 2016). While ion channels have long been established as promising therapeutic targets, the emerging understanding of metabotropic receptors in oligodendrocyte biology presents a new opportunity for therapeutic development. Together, these molecular targets may offer novel strategies to address demyelinating pathologies, such as multiple sclerosis.

Ion channels, including ligand-gated and voltage-gated channels, are already recognized as top pharmaceutical targets in the treatment of CNS disorders. In the context of demyelinating diseases, drugs modulating potassium, sodium, and calcium ion channels, such as dalfampridine, phenytoin, and pregabalin, respectively, have shown promise in mitigating symptoms and improving outcomes in preclinical models (Göbel et al., 2013; Kapoor, 2008; Schattling et al., 2014). However, despite their potential, the precise mechanisms through which these ion-channel based therapies act on the oligodendrocyte linage remain unclear. Ion channels are expressed across multiple cell types, including neurons, glia, and immune cells, complicating the ability to pinpoint their roles in oligodendrocyte-specific functions like myelination.

To move forward, further research is needed to dissect the specific roles of these channels in oligodendrocytes, particularly during the process of remyelination. This represents a critical gap in our understanding of how such targets can be leveraged to promote myelin repair in demyelinating diseases.

In contrast to the fast, transient signaling of ion channels, metabotropic receptors mediate slower, more sustained signaling cascades, making them particularly intriguing targets for long-term processes like myelin repair. Metabotropic ligands such as benztropine and clemastine (antagonist of muscarinic and histaminergic receptors, respectively) have shown efficacy in promoting OPC proliferation, differentiation, and response to injury in models of demyelination (Marangon et al., 2020). These compounds modulate oligodendrocyte lipid metabolism and have sparked interest as potential remyelinating therapies (Marangon et al., 2022; Qian et al., 2021). Additionally, adenosine receptors, particularly A_1_ and A_2A_, are crucial for the functions of OPCs and myelination, as demonstrated in various *in vitro* and *in vivo* animal models of myelination and multiple sclerosis (Cherchi et al., 2021b).

While still an emerging area, metabotropic receptors offer a promising direction for the development of therapies aimed at sustaining myelin repair. Understanding their roles in oligodendrocyte biology could open the door to interventions that modulate both fast and slow signaling to maximize remyelination.

Although the studies included in this Research Topic do not directly focus on ion channels or metabotropic receptors, they provide important insights into oligodendrocyte biology that pave the way for future investigations into these molecular targets.

For example, Moloney et al. present a dual isolation technique that facilitates the co-culture of primary neurons and oligodendrocytes from the frontal cortex of guinea pigs, offering a more accurate model for studying neuron-glia interactions. This advancement offers a platform for future studies investigating how ion channels and metabotropic receptors in oligodendrocytes respond to neuronal signals, potentially leading to better-targeted therapeutic approaches.

Additionally, advances in imaging techniques continue to enhance our understanding of myelin integrity, complementing traditional methods like electron microscopy (EM). The study by Craig et al. highlights the use of Spectral Confocal Reflectance (SCoRe) microscopy, a method that utilizes the differential refractive indices of compact CNS myelin to visualize individual myelin sheaths. This study demonstrated that SCoRe could detect differences in myelin compaction in two mouse models exhibiting myelin abnormalities without significant demyelination. These findings position SCoRe as a powerful complementary technique to EM, with promising applications for detecting subtle myelin defects. Incorporating this technique into future studies could enable the precise evaluation of myelin integrity in models of demyelination, further elucidating the role of ion channels and metabotropic receptors in oligodendrocyte function and CNS repair.

Perdaens et al. explore the role of miRNAs in regulating oligodendrocyte differentiation, a mechanism that may influence the expression or function of ion channels or metabotropic receptors, particularly in pathological conditions like multiple sclerosis. The interaction between miRNA regulation and ion/metabotropic receptor activity represents an exciting area for future research.

Similarly, Qiu et al. highlight the therapeutic potential of miRNA manipulation in spinal cord injury (SCI), demonstrating its ability to promote remyelination and repair. A significant highlight of this study is the demonstration that manipulating miRNA levels can improve functional outcomes after SCI. This work emphasizes the potential of miRNA-based therapies as a multifaceted approach to CNS repair, addressing not only the need for remyelination but also the modulation of neuroinflammation, which often complicates recovery (Qiu et al.).

Collectively, the studies included in this Research Topic provide important advancements in oligodendrocyte biology, particularly regarding miRNA regulation and cell models. While they do not directly address the role of ion channels or metabotropic receptors, they offer valuable platforms and insights that can be harnessed to explore these molecular targets in the future. By integrating these findings with current knowledge of fast and slow signaling pathways, future research can uncover new therapeutic strategies that target both ion channels and metabotropic receptors. Such an approach has the potential to revolutionize the treatment of CNS injuries and demyelinating diseases by promoting myelin repair and restoring neural function.
